# Administration of Post-intubation Analgesia and Sedation in Injured Patients in New Brunswick EDs

**DOI:** 10.7759/cureus.106773

**Published:** 2026-04-10

**Authors:** Madeline Power, Alicia Davies, Nicholas Maclean, Allison Chisholm, Susan Benjamin, Cherie-Lee Adams, Kavish Chandra

**Affiliations:** 1 Faculty of Medicine, Dalhousie Medicine New Brunswick, Saint John, CAN; 2 Trauma New Brunswick, Horizon Health Network, Saint John, CAN; 3 Department of Emergency Medicine, Dalhousie University, Saint John, CAN

**Keywords:** airway management, emergency medicine and trauma, intravenous sedation and analgesia (ivsa), major trauma, trauma center

## Abstract

Background

Despite established guidelines emphasizing the importance of post-intubation analgesia and sedation in trauma patients, adherence remains suboptimal. The goal of this study was to understand the association between appropriate post-intubation sedation and analgesia infusions received by injured and intubated adult patients in New Brunswick (NB) before and after the introduction of a provincial guideline and educational intervention.

Methods

We conducted a retrospective cohort study using data from the NB Trauma Registry, analyzing 202 ED-intubated adult trauma patients (mean age, 46 years; 78% male) treated at Level 1-3 and 5 centers. The primary outcome was the proportion of patients receiving both analgesia and sedation prior to the creation and implementation of the Trauma NB consensus statement, Adult Rapid Sequence Intubation and Post-Intubation Analgesia and Sedation for Major Trauma Patients, and the subsequent educational intervention. We chose 2017 as the pre-intervention period, 2019 as the post-intervention period, and 2021 as the follow-up period. Secondary outcomes included whether no infusions were given, only analgesia was given, or only sedation was given.

Results

A total of 202 intubated adult trauma patients were included: 62 pre-intervention (2017), 72 post-intervention (2019), and 68 at follow-up (2021). The cohort had a mean age of 46 years and was predominantly male (78%). Motor vehicle collisions (51.5%) and falls (20.3%) were the most common mechanisms of injury, with a mean pre-intubation Glasgow Coma Scale score of 9. The proportion of patients receiving both analgesia and sedation increased significantly from 22.6% (95% CI, 13.8-34.5%) in 2017 to 61.1% (49.6-71.6%) in 2019 and 73.5% (61.9-82.6%) in 2021, representing a 3.3-fold improvement (p < 0.05). Omission of both therapies declined from 41.9% (30.4-54.3%) pre-intervention to 16.7% (9.6-27.1%) post-intervention and 8.8% (3.8-18.3%) at follow-up (p < 0.05). Sedation-only use decreased from 30.7% (20.5-43.0%) to 13.9% (7.5-23.9%) and 8.8% (3.8-18.3%), while analgesia-only use remained uncommon (4.8-8.8%).

Conclusions

Province-wide guideline implementation and educational intervention were associated with improved adherence to evidence-based post-intubation pharmacotherapy, with sustained effects at three-year follow-up. These findings suggest that standardized protocols and educational interventions may be effective tools for improving the quality of trauma airway management.

## Introduction

Critically injured patients requiring emergency airway management face substantial physiological and psychological stress, underscoring the importance of effective post-intubation pharmacological support. Rapid sequence intubation (RSI), defined as the rapid administration of sedative and paralytic agents followed by endotracheal intubation, remains the cornerstone of emergency airway management [1-3]. When performed adequately, RSI optimizes intubation conditions to maximize first-pass success rates [4]. Nevertheless, this procedure demands meticulous peri-intubation management to mitigate adverse events, catecholamine-mediated sympathetic surge, and hemodynamic instability [5-8].

Effective post-intubation analgesia and sedation serve as critical safeguards against these risks, reducing not only acute pain but also short- and long-term sequelae such as awareness of paralysis and post-traumatic stress disorder [5-9,10]. Despite these benefits, an evidence-to-practice gap still exists. Recent data from a national study in the USA revealed that fewer than 25% of intubated trauma patients receive guideline-concordant analgesia within the critical first hour [9]. Another study reported that over 80% of patients in intensive care units recall pain or discomfort associated with the endotracheal tube [11]. This gap generates measurable harm: inadequately controlled pain amplifies catecholamine-driven hemodynamic instability during resuscitation, and competing clinical priorities (e.g., simultaneous hemorrhage control and neurological assessment) often displace pharmacological optimization in high-acuity ED environments [5].

In New Brunswick (NB), Trauma NB coordinates provincial trauma care, education, and quality improvement. Recognizing inconsistencies in RSI and post-intubation management, particularly the underuse of analgesia and variable sedation practices, the program developed the Adult Rapid Sequence Intubation and Post-Intubation Analgesia and Sedation for Major Trauma Patients consensus statement (2018, updated in 2025) [12]. This initiative provided free, open-access, evidence-based protocols, checklists, and educational resources to EDs across all trauma center levels (1-5) as an educational intervention. Furthermore, these guidelines and checklists were integrated into Trauma NB’s Mobile Simulation program, a team-based medical simulation that travels to Level 1-5 trauma centers throughout NB. Post-case debriefing was also offered to trauma teams following the management of intubated trauma patients.

The impact of Trauma NB’s intervention on analgesia and sedation practices in intubated trauma patients remains unquantified. This study evaluated the association between the implementation of the consensus statement and subsequent educational intervention and appropriate post-intubation pharmacotherapy at pre-intervention (2017), post-intervention (2019), and follow-up (2021) time points.

The results of this article were previously presented at the Trauma Association of Canada annual meeting in Halifax, Nova Scotia, Canada, as an oral presentation on May 9, 2024.

## Materials and methods

Eligibility criteria

We conducted a retrospective cohort study using data from the NB Trauma Registry to evaluate the impact of the 2018 provincial consensus statement and educational intervention on post-intubation analgesia and sedation practices [12]. The study included all adult trauma patients (≥19 years) who underwent ED intubation during three periods: pre-intervention (2017), post-intervention (2019), and follow-up (2021). Patients were excluded if they received cardiopulmonary resuscitation, died in the ED, were extubated, or had an ED length of stay <30 minutes post-intubation, as these patients’ pharmacological management was taken over by critical care and/or anesthesiology teams.

Data collection and variables

The NB Trauma Registry collects data on all patients who present to or are transferred to Level 1, 2, and 3 centers and are admitted within the province of NB, Canada, with an Injury Severity Score (ISS) >12 for blunt injuries and >9 for penetrating injuries. Patients are entered into the registry retrospectively by trauma resource nurses through structured chart reviews using standardized definitions and ICD codes.

Intervention

Trauma NB coordinates provincial trauma care, education, and quality improvement. Recognizing inconsistencies in RSI and post-intubation management, the program developed the Adult Rapid Sequence Intubation and Post-Intubation Analgesia and Sedation for Major Trauma Patients consensus statement (2018, updated in 2025) [12]. This initiative provided free, open-access, evidence-based protocols, checklists, and educational resources to EDs across all trauma center levels (1-5) as an educational intervention. These guidelines and checklists were also integrated into Trauma NB’s Mobile Simulation program, a team-based medical simulation that travels to Level 1-5 trauma centers throughout NB. In addition, post-case debriefing was offered to trauma teams following the management of intubated trauma patients.

Outcome measures

The primary outcome was the proportion of patients who received both sedation and analgesia infusions following intubation. The secondary outcome was the proportion of patients receiving sedation only, analgesia only, or neither infusion. Ketamine infusions were classified as both analgesic and sedative per guideline specifications. Covariates included demographics (age and biological sex), injury characteristics (mechanism and Glasgow Coma Scale (GCS) score), facility trauma level (1-5), and discharge disposition. Only patients with complete data were included in the final analysis.

Statistical analysis

Proportions of patients meeting outcome criteria were compared across time points using a 3 × 2 chi-square test of independence, with the null hypothesis stating equal proportions across years. Where significant differences were detected (p < 0.05), post hoc pairwise comparisons with the Benjamini-Hochberg correction identified specific temporal differences. Fisher’s exact test was substituted when >80% of contingency table cells had expected frequencies <5. Analyses were performed in R (version 4.3.1; R Foundation for Statistical Computing, Vienna, Austria), with visualizations created using Prism (version 9.3.0; GraphPad Software, San Diego, California, USA). A power analysis (Cramer’s V = 0.2, α = 0.05, power = 0.8) determined that 197 patients would suffice to detect small-to-medium effects.

Ethical considerations

This study received approval from Horizon Health Network’s Human Research Protection Program and REB (file #101916), with a waiver of consent granted according to Tri-Council Policy Statement 2 Article 5.5A [13].

## Results

**Figure 1:**
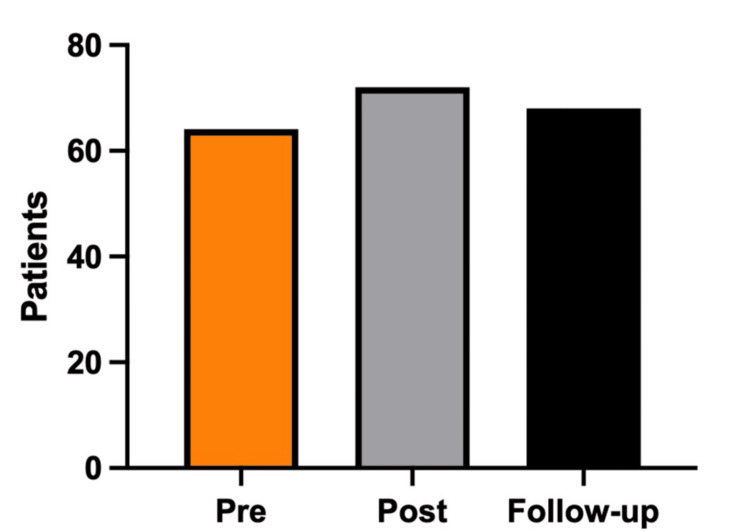
Intubated ED trauma patients

A total of 202 intubated trauma patients met the inclusion criteria across the study periods: pre-intervention (2017, n = 62), post-intervention (2019, n = 72), and follow-up (2021, n = 68) (Figure 1). The study population was predominantly male (78.2%), with a mean age of 46 years (range, 19-95; Table 1). Patients were most frequently treated at Level 3 trauma centers (40.6%), followed by Level 5 (21.8%) and Level 2 (21.3%) centers. Motor vehicle collisions (51.5%) and falls (20.3%) were the leading mechanisms of injury. The mean GCS score prior to intubation was 9 (range, 3-15), reflecting moderate-to-severe injury acuity.

**Table 1:** Patient demographics and clinical characteristics * Other mechanisms for overall years: assault (6.4%), burns (5.9%), self-harm (8.4%), sports (1.0%), non-motor (2.0%), and unknown (1.5%) GCS, Glasgow Coma Scale

Characteristic	Overall (N = 202)	2017 (n = 62)	2019 (n = 72)	2021 (n = 68)
Age, years (range)	46 (19-95)	45 (19-94)	44 (21-95)	48 (19-82)
Biological sex				
Male	158 (78.2%)	46 (74.2%)	57 (79.2%)	55 (80.9%)
Female	44 (21.8%)	16 (25.8%)	15 (20.8%)	13 (19.1%)
Trauma center level				
Level 1	33 (16.3%)	7 (11.3%)	18 (25.0%)	8 (11.8%)
Level 2	43 (21.3%)	13 (21.0%)	16 (22.2%)	14 (20.6%)
Level 3	82 (40.6%)	26 (41.9%)	29 (40.3%)	27 (39.7%)
Level 5	44 (21.8%)	16 (25.8%)	9 (12.5%)	19 (27.9%)
GCS, mean ± SD	9 ± 5	9 ± 5	8 ± 4	9 ± 4
Injury mechanism				
Motor vehicle collision	104 (51.5%)	31 (50.0%)	37 (51.4%)	36 (52.9%)
Fall	41 (20.3%)	11 (17.7%)	17 (23.6%)	13 (19.1%)
Other*	56 (28.2%)	20 (32.3%)	18 (25.0%)	19 (28.0%)

**Figure 2:**
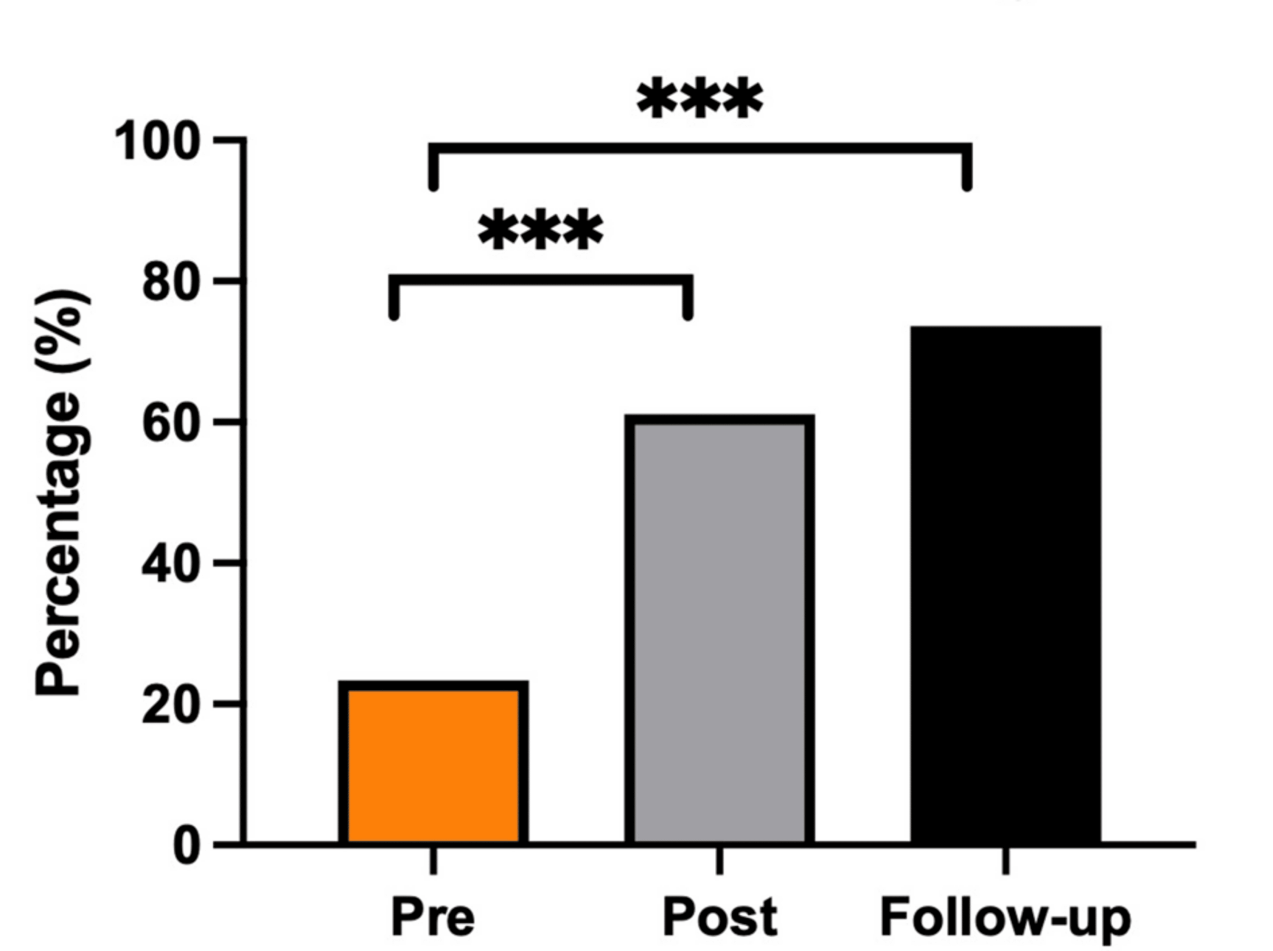
Intubated ED trauma patients receiving both sedation and analgesia infusions *** p < 0.05, post hoc pairwise comparison

**Figure 3:**
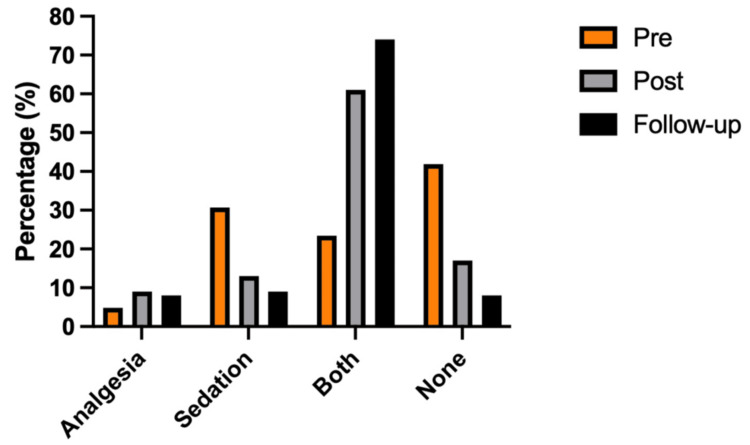
Intubated ED trauma patients in the pre-, post-, and follow-up periods

The proportion of patients receiving both analgesia and sedation increased significantly from 22.6% (95% CI, 13.8-34.5%) in 2017 to 61.1% (49.6-71.6%) in 2019 and 73.5% (61.9-82.6%) in 2021, representing a 3.3-fold improvement (p < 0.05; Figure 2). Omission of both therapies declined from 41.9% (30.4-54.3%) pre-intervention to 16.7% (9.6-27.1%) post-intervention and 8.8% (3.8-18.3%) at follow-up (p < 0.05; Figure 3). Sedation-only use decreased from 30.7% (20.5-43.0%) to 13.9% (7.5-23.9%) and 8.8% (3.8-18.3%), while analgesia-only use remained uncommon (4.8-8.8%; Figure 3).

## Discussion

Through a longitudinal evaluation of 202 intubated trauma patients, this study demonstrates that Trauma NB’s 2018 consensus statement and subsequent educational intervention were associated with a sustained 3.3-fold increase in guideline-concordant post-intubation analgesia and sedation practices across NB’s trauma centers. The intervention’s success in reducing omissions of both sedative and analgesic infusions from 41.9% to 8.8% highlights the role of standardized, system-wide protocols in closing critical evidence-practice gaps that persist globally. Our findings underscore that coordinated provincial educational initiatives can achieve durable practice changes where fragmented, institution-specific efforts often falter.

Previous studies

The magnitude and sustainability of the observed improvement (73.5% adherence by 2021) exceed rates reported in a comparable ED study, which showed that fewer than 25% of intubated trauma patients received guideline-concordant analgesia within the critical first hour [9]. This divergence likely stems from our multimodal educational approach, which integrated evidence-based guidelines, checklists, interprofessional collaboration, and post-case debriefing across all trauma care levels, as opposed to single-center interventions that initially focused solely on order sets or nursing protocols [13].

While this study did not address the timing of sedation and analgesic infusion initiation, earlier initiation is associated with better patient outcomes. Among tertiary EDs in Australia, one study reported a median time to infusion of seven minutes [14]. Another study showed that paralytic choice is associated with the timing of infusion initiation, with longer initiation times in patients who received long-acting paralytics [15]. This is likely, in part, owing to patient agitation or ventilator dyssynchrony alerting clinicians that induction sedatives have worn off and patients are now inadequately sedated and treated for pain.

Strengths and limitations

Key strengths include our province-wide scope (encompassing Levels 1-5 trauma centers) and standardized data collection via the NB Trauma Registry, which minimized inter-facility variability. Nevertheless, several limitations warrant consideration. First, as a retrospective cohort study, unmeasured confounders (e.g., variations in clinician experience) may persist. Second, temporal confounding may exist, as the study spans a broad period; trends in emergency medicine may account for some of the improvement. While we documented medication administration, the absence of dosing data precludes assessment of whether regimens met weight-based therapeutic thresholds. We did not quantify sedation depth, which is clinically relevant, as adequate sedation depth facilitates stabilization of critically ill patients; however, data suggest that early deep sedation may be associated with worse outcomes [16]. Finally, the potential for undetected awareness of paralysis, which occurs in 0.005-0.15% of anesthesia cases and 2.6% of ED patients, remains a concern when evaluating sedation adequacy [10,17].

Clinical implications

Our findings have direct implications for emergency airway management, supporting the routine integration of standardized protocols and educational interventions to improve early post-intubation care. Time and again, studies demonstrate that the publication of guidelines alone has a limited effect on changing clinical practice. One quality improvement study showed a near doubling in the frequency of patients receiving sedation and analgesic infusions after ED intubation following physician and nursing education and adoption of an electronic order set [18].

Research implications

We excluded 2020 data due to COVID-19 disruptions, which, while necessary, limits insight into pandemic-related impacts on guideline adherence. Retrieving data from that time period may provide insight into how to prepare for future resource-straining events, given the ED resource constraints during that period. Future work should focus on pandemic disruptions, long-term maintenance of the trends seen in our study, timing of infusion initiation, sedation depth, and associated patient outcomes.

## Conclusions

Our findings establish that implementing a standardized consensus statement along with educational interventions was associated with sustained improvements in post-intubation pharmacotherapy across NB’s trauma system. These findings highlight the role of coordinated, system-level approaches for translating evidence into practice, offering a replicable model for trauma systems seeking to bridge persistent care gaps. Future research should examine how these practice changes affect patient-centered outcomes.
